# Green Synthesis of CS-TiO_2_ NPs for Efficient Photocatalytic Degradation of Methylene Blue Dye

**DOI:** 10.3390/polym14132677

**Published:** 2022-06-30

**Authors:** Mohammad BinSabt, Varsha Sagar, Jagpreet Singh, Mohit Rawat, Mohamed Shaban

**Affiliations:** 1 Chemistry Department, Faculty of Science, Kuwait University, P.O. Box 5969, Safat 13060, Kuwait; mohammad.binsabt@ku.edu.kw; 2 Department of Nanotechnology, Sri Guru Granth Sahib World University, Fatehgarh Sahib 140406, India; varshasagar1997@gmail.com; 3 Department of Chemical Engineering, Chandigarh University, Gharuan, Mohali 140413, India; 4 University Centre for Research and Development, Chandigarh University, Gharuan, Mohali 140413, India; 5 Nanophotonics and Applications (NPA) Lab, Faculty of Science, Physics Department, Beni-Suef University, Beni-Suef 62514, Egypt; 6 Biochemistry Department, Faculty of Science, Beni-Suef University, Beni-Suef 62521, Egypt

**Keywords:** green synthesis, photocatalysis, *cannabis sativa* (bhang), methylene blue dye, TiO_2_ NPs

## Abstract

The development of a non-malignant and sustainable treatment approach for eradicating mephitic organic dyes from freshwater resources is a daunting task. In a similar vein, the current work investigates the mitigation of methylene blue (MB) dye utilizing titanium dioxide nanoparticles (CS-TiO_2_ NPs) synthesized using *cannabis sativa* (bhang) leaf extract via a greener approach. The CS-TiO_2_ NPs are well characterized through XRD, FE-SEM, HR-TEM, UV-Vis spectroscopy, FTIR spectroscopy, and EDS spectroscopy. Microscopic studies confirm that the average particle size distribution of the individual particles was found to be in the range of 12.5 ± 1.5 nm, whereas the average size of the CS-TiO_2_ NPs aggregates is 24.5 ± 11.5 nm. Additionally, the synthesized CS-TiO_2_ NPs manifested remarkable photocatalytic degradation potential against methylene blue dye with a degradation efficiency of 98.2% and an apparent rate constant of 0.0398 min^−1^. As a result, this research offers a green/sustainable alternative for water purification.

## 1. Introduction

Water is one of the most important components for all living beings on the planet to survive. In recent decades, there has been a constant rise in population, worldwide economic expansion, and reliance on certain businesses that pollute the groundwater. Due to the shortage of groundwater sources, eradication of water contaminants is among the most critical hurdles for the research world, since this process necessitates the creation of more sophisticated technologies or the improvement of presently utilized materials or procedures [1,2]. Pollutants include anything that tends to vary in chemical or physical qualities, as well as additives. Chemical, physical, radioactive, organic, or inorganic waste are examples of pollutants. Such contaminants pose a serious concern to aquatic and wildlife since they inflict severe harm to their neurological systems, leading to the development of cancerous disorders [2].

Dyes and related hydrocarbons, which are among the most major and hazardous contaminants in water, are connected to population growth owing to their industrial usage. Dyes are commonly associated with the textile, printing, and paper industries, but they are also applied to foodstuffs, healthcare, sports, and aesthetics [3]. Because dyes are organic compounds with high solubility in water, recovering colors from polluted water using traditional techniques is challenging. Given that the loss of water tainted with dyes surpasses 15%, the textile sector consumes a massive volume of water, maybe exceeding 800 thousand tons per year [4,5].

Considering the fact that dyes are extremely poisonous, there are several health risks associated with their consumption or use. Carcinogens exist in pigments. They are highly toxic to aquatic species because they restrict the accessibility of liquid to sunlight, lowering metabolic rates and oxygen saturation in the seawater [5]. Methylene blue (MB) is a cationic dye that is utilized in a wide range of chemical, biological, medicinal, and other applications. Diarrhea, discomfort, anemia, and elevated blood pressure are all common side effects of MB usage [6].

Coagulation, adsorption, biodegradation, membrane process, activated sludge treatment process (ASTP), and advanced oxidation process (AOP) are some of the methods that have been used to eliminate these harmful colors from industrial effluents. Each of these procedures has its own set of advantages and disadvantages, which are chosen based on the requirements [7,8,9]. For instance, most effluent treatment plants use the traditional ASTP because of its inexpensive cost; nevertheless, this technology is ineffective at eliminating hazardous organic dyes [10]. However, alternative physicochemical treatment procedures, such as coagulation and adsorption, need a considerable amount of chemicals and thus are not ecologically friendly [11]. Whereas membrane processes, such as membrane bioreactors, are effective, they are inefficient in terms of energy, and have a high operational cost [12].

The AOPs, for example, offer tremendous promise in the treatment of dye-based textile effluents because they can degrade soluble organic pollutants from wastewaters [13].

Heterogeneous photocatalytic degradation-based AOPs are attractive solutions for organic dye degradation because they are more successful than other AOPs owing to the inexpensive cost of semiconductor-based photocatalysts and high effectiveness in mineralizing a range of organic dyes [14]. Semiconducting photocatalysts based on nanoparticles (NPs) have a huge potential for AOP-based wastewater treatment. Nanomaterials, such as nanocrystalline transition-metal oxides, have a considerable standard of reactivity, a wide range of functionalization adaptability, a huge surface area, and other size-dependent features [15,16,17]. Numerous semiconducting NPs, such as ZnO, CuO, TiO_2_, NiO, SnO_2_, and others, have been used as photocatalysts for the removal of organic dyes [18,19,20,21,22,23]. Because of their stability, cheap cost, and optical absorption in the ultraviolet range, TiO_2_-based photocatalysts have been extensively used for the degradation eradication of organic dyes.

The chemical preparation of TiO_2_ NPs involves specific conditions; high temperature and expensive and toxic chemicals also limit TiO_2_ applications owing to serious eco-toxicological concerns. The green synthesis of TiO_2_ NPs using sustainable components such as plant extracts, microorganisms, and enzymes is now becoming increasingly prevalent due to its simplicity, cost effectiveness, greener, and minimal dangerous nature. Furthermore, the use of plant extracts to make semiconducting TiO_2_ NPs has piqued attention because it avoids the use of chemicals and other contaminants while also enhancing the environmental suitability of the remediation process. To date, few studies have explored the utilization of green TiO_2_ NPs for dye degradation [13,24,25]. Nonetheless, there is a continual impulse for an ecologically friendly TiO_2_ NP preparation. In this approach, organic components cap the prepared NPs to avoid agglomeration, which is known as steric stabilization.

In this regard, the current study uses *cannabis sativa* plant extract to synthesize TiO_2_ NPs in a green manner. *Cannabis sativa*, generally known as hemp, is a plant belonging to the cannabinaceae family that contains the pain-relieving ingredient THC (delta-9 tetrahydrocannabinol). *Cannabis sativa* contains medicinal benefits that have been used for hundreds of years in numerous cultures, including the treatment of pain, asthma, sleeplessness, depression, and lack of appetite [26]. In addition, the prepared CS-TiO_2_ NPs were used as a photocatalyst for the eradication of the methylene blue dye.

## 2. Materials and Methods

### 2.1. Materials

Parswanath Dye Stuff Industries in Ahmedabad, India, provided a methylene blue dye. All the chemicals and reagents used for the preparation of CS-TiO_2_ NPs were purchased from Merck India Ltd., Mumbai, India. The leaves came from a local university park.

### 2.2. Preparation of Extract

About 10 gm of *cannabis sativa* (bhang) leaves were thoroughly cleaned with water to eradicate any tainted substance before being air-dried. The air-dried leaves were then chopped into fine bits and heated for one hour in 100 mL distilled water. A light brown solution was attained using the filtration method (Whatman filters with 1.5 μm pore size) by separating from residual particulate matter.

### 2.3. Preparation of CS-TiO_2_ NPs

In total, 6 mL of 0.5 M Titanium isopropoxide [Ti{OCH(CH_3_)_2_ }_4_] suspension was pipetted to 40 mL of purified CS extract in a 1:1 (*v*/*v*) ratio with constant stirring at room temperature (RT) for obtaining CS-TiO_2_ NPs using the green technique. The reduction in metallic ions (Ti^4+^) in the solution caused a visible color change from translucent to whitish brown within 10 min of mixing reactant and leaves extract, revealing the synthesis of CS-TiO_2_ NPs. In total, 6 mL ammonia was drop-wisely introduced to the NP suspension under continuous stirring at room temperature to create the NPs in precipitate form. Filtration was used to isolate the resultant NP precipitates from the mixture. Then, the resulting solution was filtered twice or three times with filter paper before being rinsed with ethanol to confiscate ionic contaminants. After that, the precipitates were air-dried and calcined in a muffle furnace at 400 °C for 4 h to obtain the fine white powder of CS-TiO_2_ NPs, which was then crushed in a crystal mortar pestle.

### 2.4. Characterization Techniques

The crystalline nature and lattice parameters of prepared CS-TiO_2_ NPs were determined using PANalytical’s X’Perto Powder X-ray Diffraction (PXRD). The geometry and size of the particle were demonstrated through Hitachi HF-3300 Transmission Electron Microscope (Hitachi, Tokyo, Japan). The optical absorption spectra of the samples were recorded using a Shimadzu UV-2600 (Shimadzu, Kyoto, Japan) research-grade spectrophotometer. The carbonaceous vibrational characteristics of CS-TiO_2_ were determined using a Bruker Alpha Fourier Transformed Infrared (FTIR) spectrophotometer (Bruker, Billerica, MA, USA). The elemental composition of the prepared CS-TiO_2_ NPs was determined using Energy Dispersive Spectroscopy (EDS) analysis supplied by Oxford Instruments.

### 2.5. Photocatalytic Activity

In the case of a normal dye decomposition, 5 mM of methylene blue dye was prepared in 100 mL of deionized water and 1 mg of prepared CS-TiO_2_ NPs added to this solution. For maximum dye adsorption, the mixture was maintained in the darkness for 16 h. The solution was then subjected to UV light (UV-B, λ = 280–315 nm). After every 10 min of UV irradiation, 5 mL of the mixture was withdrawn and centrifuged to recover the photocatalyst. A UV-Vis spectrophotometer was used to check the dye content in the supernatant containing transparent dye solution. The intensity of the dye gradually decreased with irradiation time, implying that the chemical arrangement of the dye was breaking up. The photocatalytic measurements were carried out at room temperature (20 °C).

## 3. Results and Discussion

### 3.1. Optical Study

**Figure 1:**
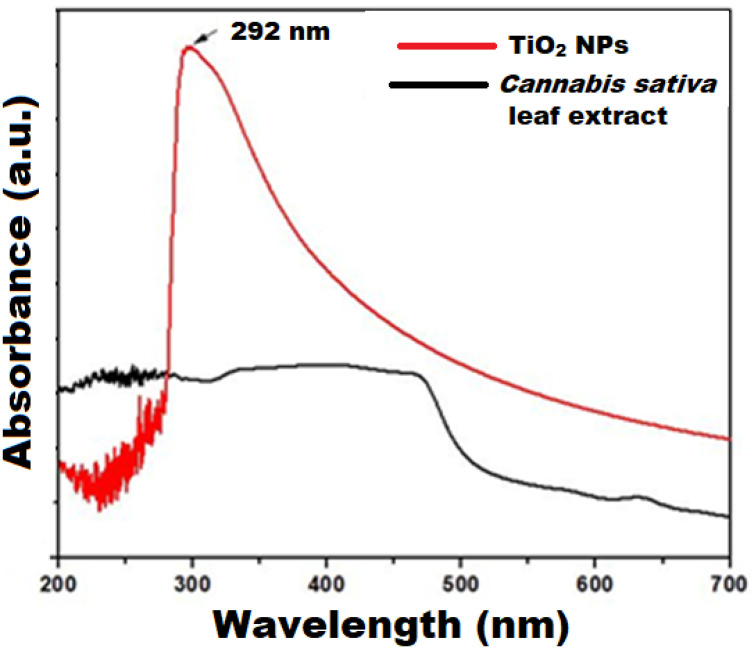
UV-vis spectra of *cannabis sativa* leaf extract and synthesized CS-TiO_2_ NPs.

The particle size and shape are the determining factors for the optical absorption properties of NPs. To explain this behavior, UV-Vis spectra have been recorded for the *cannabis sativa* leaf extract and as-synthesized CS-TiO_2_ NPs as shown in Figure 1. For *cannabis sativa* leaf extract, the absorption is weak and no peaks are detected. For the as-synthesized CS-TiO_2_ NPs, a significant absorption peak was detected at 292 nm, providing base confirmation for the greener approach to synthesizing CS-TiO_2_ NPs. Nevertheless, the absorption band of the obtained biogenic CS-TiO_2_ NPs (292 nm) was significantly bluer than that of bulk TiO_2_ (380 nm), indicating the quantum confinement characteristic of NPs. Moreover, the maximum absorption band around 300 nm corresponds to the anatase phase of TiO_2_ NPs [27]. As the particle size is lowered, the absorption edge shifts to a lower wavelength, which can be described through the quantum confinement process. The optical band gap was obtained using the maximum absorption wavelength of CS-TiO_2_ NPs to be 4.24 eV, which is greater than the bulk titanium (3.2 eV).

### 3.2. FTIR Spectrum

**Figure 2:**
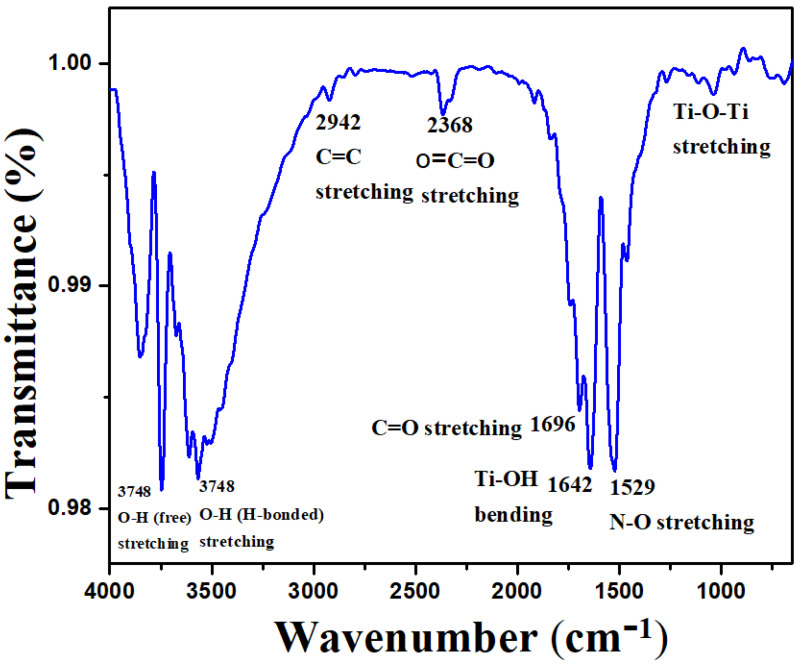
FTIR spectra of synthesized CS-TiO_2_ NPs by *cannabis sativa* leaf extract.

As shown in Figure 2, the FTIR spectrum of CS-TiO_2_ NPs derived from *cannabis sativa* leaf extract was investigated in the range of 4000–500 cm^−1^. As a result of inter-atomic vibrations, metal oxides generally have bands in the fingerprint region below 1000 cm^−1^ due to Ti–O and Ti–O–Ti bending vibrations [28]. The distinct band at 685 cm^−1^ is most usually related to the anatase phase and is attributed to the Ti–O vibration. The characteristics band of CS-TiO_2_ NPs at 1642 cm^−1^ is attributed to the stretching vibration of Ti–OH [29]. The band at 1696 cm^−1^ is ascribed to the C=O stretching vibration. The bending vibrations of N–O stretching are depicted by the sharp band at 1529 cm^−1^, whereas the bands at 2368 cm^−1^ and 2942 cm^−1^ are ascribed to O=C=O and C=C vibrations. The bands within the 4000–3500 cm^−1^ region inferred the H-bonded stretching and the free stretching of O-H vibrations [28,30]. According to established literature, *cannabis sativa* extract has a larger quantity of phenolics and flavonoids, which are essential for NP reduction, capping, and stability. It is noticeable from the FTIR spectra of TiO_2_ NPs that phytochemicals (phenols, flavonoids, cannabinoids, polysaccharides, water-soluble biomolecules, etc.) present in *cannabis sativa* extract are vital for bioreduction. As a result, proteins and other biomolecules containing -OH moiety attach to the NP’s surface and inhibit agglomeration [31,32,33].

### 3.3. XRD Analysis

**Figure 3:**
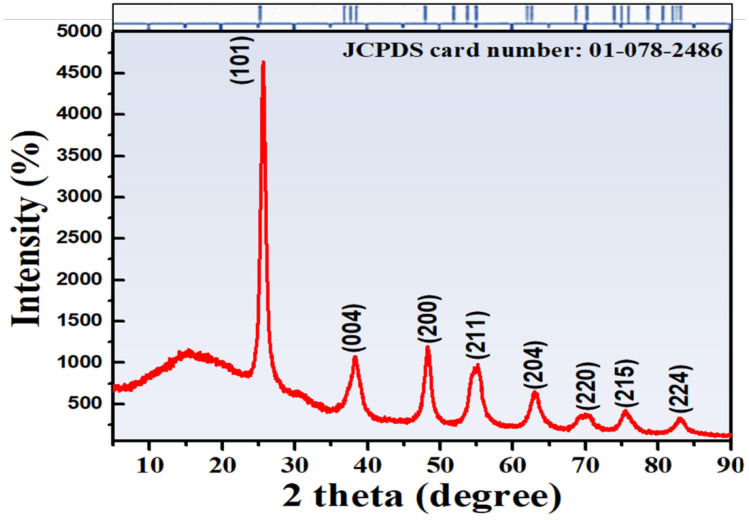
XRD spectrum for crystal structure investigation provided with JCPDS 01-078-2486 card.

Figure 3 shows the XRD spectrum of CS-TiO_2_ NPs. The established XRD peaks observed at 2Ɵ values of 25.3°, 38.2°, 48.3°, 54.8°, 62.8°, 70.0°, 75.3°, and 82.1° revealed the tetragonal structure of anatase CS-TiO_2_ NPs with corresponding (101), (112), (200), (105), (204), (220), (215), and (224) planes, respectively. The measured peaks resemble the standard tetragonal structure of anatase TiO_2_ (JCPDS Card No. 01-078-2486). As shown in Figure 3, the JCPDS Card No. 01-078-2486 fits all peaks, indicating that no contaminants or mismatched peaks have been found.

The average crystallite size can be determined from the XRD pattern using the Debye–Scherrer formula as given in Equation (1):

D = Kƛ/βcosθ = 0.89ƛ/βcosθ(1)

where D is the mean crystallite size, K is Scherrer’s constant (0.94), ƛ is the wavelength, β is the full-width half-maximum, and θ is Bragg’s angle. The average crystallite size was calculated to be 8 nm using Equation (1).

### 3.4. FE-SEM Analysis

**Figure 4:**
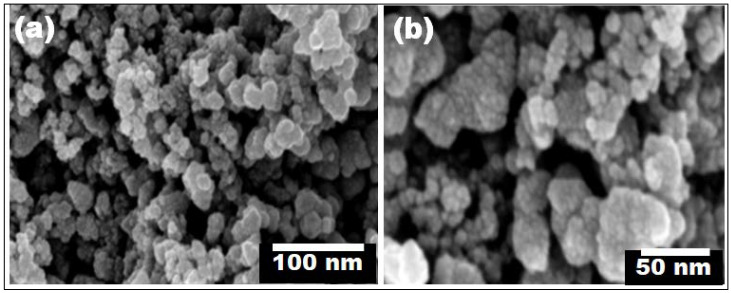
(**a**,**b**) SEM images of the green synthesized CS-TiO_2_ NPs at two different magnifications.

The information about the morphological characteristics of the obtained particles was provided by the FE-SEM micrographs. Figure 4 shows FE-SEM images taken at two different magnifications for the precipitated CS-TiO_2_ NPs after calcination at 400 °C for 4 h without ageing. It was confirmed that the particles were almost spherical in shape. As demonstrated in Figure 4, a considerable number of small and distinct CS-TiO_2_ NPs were found, compared to a comparatively limited number of large-sized agglomerated NPs. The average particle size of the individual CS-TiO_2_ NPs is 14 ± 3 nm.

### 3.5. HR-TEM Analysis

**Figure 5:**
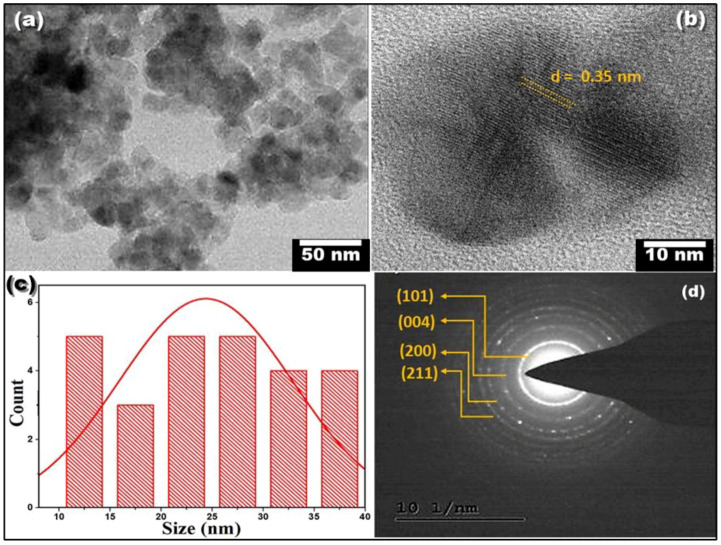
Spectroscopic studies: (**a**,**b**) HR-TEM images, (**c**) particle size distribution histogram, and (**d**) SAED pattern of CS-TiO_2_ NPs.

Individual CS-TiO_2_ particle calcination at 400 °C for 4 h is shown in the HR-TEM images, Figure 5a,b at two different magnifications. CS-TiO_2_ NPs have a consistent spherical form. Figure 5b shows a HR-TEM micrograph of anatase TiO_2_ NPs at a greater magnification. The anatase form of biogenic TiO_2_ (101) NPs with a fringe width of 0.35 nm was verified. i.e., the d-spacing value was found to be 0.35 nm. Figure 5c shows the histogram of the particle size distribution. The average particle size distribution of the individual particles was found to be in the range of 12.5 ± 1.5 nm, whereas the average size of the aggregates is 24.5 ± 11.5 nm. The crystalline structure of biogenic CS-TiO_2_ NPs was further verified by a SAED pattern, Figure 5d, with bright spherical rings corresponding to planes (101), (004), (200), (105), (211), (204), (220), and (215), respectively, of the anatase crystal [34,35]. The lattice fringes clearly show that the particles are crystalline with an anatase phase, which is supported by the XRD and SAED results. Hence, the brighter spot and strong rings in the SAED pattern indicate that the green method created highly crystalline titanium dioxide NPs.

### 3.6. Photocatalytic Activity Test of CS-TiO_2_

**Figure 6:**
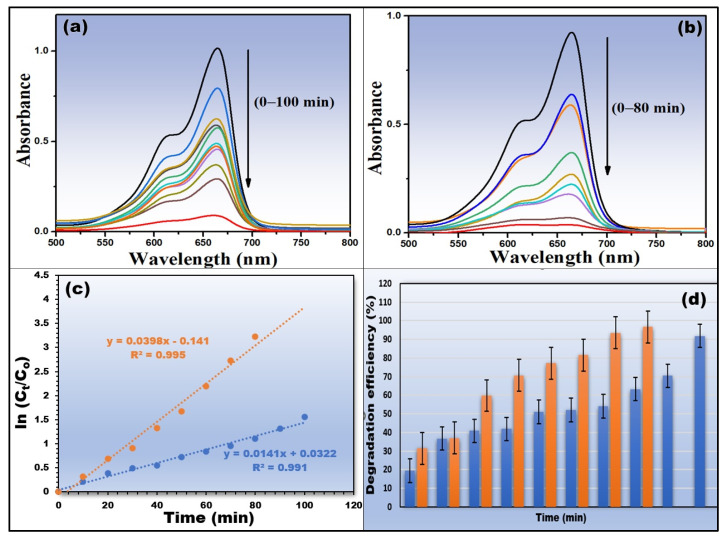
Photocatalysis study: (**a**,**b**) UV–Vis spectra of photocatalytic degradation of MB dye using 5 mg and 10 mg of green synthesized CS-TiO_2_ NPs, (**c**) pseudo-first-order kinetic study, and (**d**) histogram of degradation efficiency.

Due to its resistive nature, methylene blue dye is said to be a common hazard in the wastewater produced by textile factories, among other things. The photocatalytic capabilities of obtained CS-TiO_2_ NPs were tested via observing the decay of MB dye in direct sunlight. To obtain the MB dye solution, 5 mg of MB was dissolved in 100 mL DI water. The prepared CS-TiO_2_ was immersed in MB dye solution with a pH 9. To ensure dye adsorption/desorption equilibrium, the solution was agitated for a few hours. After exposing the system to sunlight for 20 min, a 2 mL aliquot was taken for photocatalytic analysis at the MB dye’s optimum absorbance peak of 664 nm. Figure 6a,b display the plots of photodegradation of MB dye using 5 mg and 10 mg dosages of green synthesized CS-TiO_2_ NPs for varying exposure times. Solar irradiation causes the chemical structure of MB dye to break down, and the MB degradation percentage reached 92.2% within a reaction period of 100 min using a catalyst dose of 5 mg (Figure 6a), whereas the observed removal percentage of MB dye is 98.2 percent within 80 min using a dose of 10 mg (Figure 6b).

Figure 6c shows the plots of the pseudo-first-order degradation kinetics at 5 mg and 10 mg catalyst doses [36,37,38,39]. This figure reveals linear relationships with correlation coefficients > 0.99 as shown in Table 1, showing that the photocatalytic reaction has pseudo-first-order degradation kinetics. The apparent rate constants (K_app_ (min^−1^)) for the photocatalytic activity conducted at dose values of 5 mg and 10 mg, as derived from the kinetic plots, Figure 6c, are 0.0141 min^−1^ and 0.0398 min^−1^, respectively. Figure 6d illustrates a histogram of degradation efficiency versus the exposure time, which shows an increase in the degradation efficiency when the exposure time is increased. This figure provided error bars of triplicate measurements.

Table 2 shows the performance of our designed photocatalyst relative to the previously reported TiO_2_-based photocatalysts [35,36,37,38,39,40,41,42,43,44,45,46]. Our design showed higher photocatalytic degradation% within a short time relative to the reported data in references [36,41,43,44,46]. Additionally, using a lower catalyst dose than that reported in references [35,37,38,39,40,42,45], showed high catalytic efficiency.

### 3.7. Mechanism of Dye Degradation

**Figure 7:**
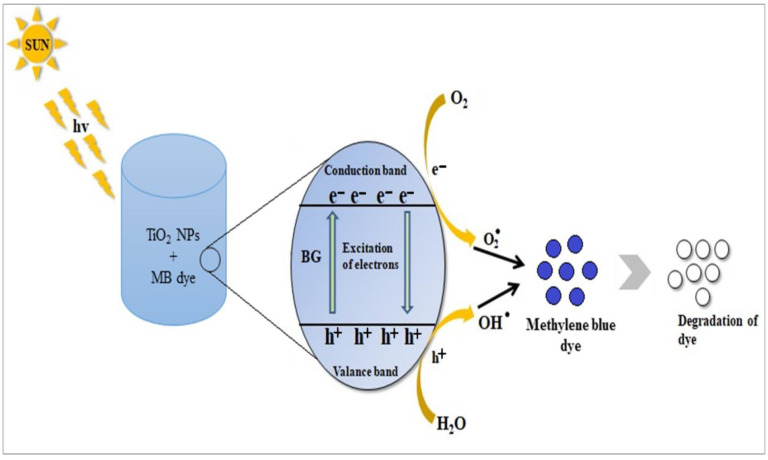
The method of photocatalytic degradation of MB under continuous solar irradiation is depicted in this diagram.

Light-dependent free radical production on the surface of CS-TiO_2_ NPs results in their photocatalytic activity. The photo-oxidation process begins with the light-dependent activation of CS-TiO_2_ NPs, which produces superoxide (O^2−^) ions and hydroxyl (OH) radicals, and these reactive chemicals interact with analytes to catalyze deterioration. In short, electron-hole pairs are formed when sunlight strikes the surface of CS-TiO_2_ NPs. Superoxide radical (O^2−^) anion is formed when photogenerated electrons in the conduction band react with oxygen molecules (O_2_). The positive holes attack the water to form hydroxyl radicals OH in the same stride. These potent oxidation reactions, O^2−^ and OH radicals, are accountable for the photo-oxidation of dye [46]. Figure 7 depicts one probable dye degradation mechanism.

*Cannabis sativa* (bhang)-mediated CS-TiO_2_ absorbs an appropriate frequency of irradiation. The electron is stimulated to the conduction band and a positive charge (hole) is produced in the lower band of CS-TiO_2_ [39]:

CS-TiO_2_ + h*v*_(photon)_→e^−^ _(C.B.)_ + h^+^_(V.B.)_ (2)

The participation of these created charged carriers on the surface caused several reactions:

- Oxidation: h^+^_(V.B.)_ + H_2_O→H^+^ + OH.
- Reduction: e^−^ _(C.B.) +_ O_2 (adsorbed)_→O_2_^−^.
- Powerful oxidizing substances destroyed the MB dye: O_2_^−^ or OH + MB)→intermediates (peroxylated or hydroxylated)→H_2_O + CO_2_ + innocuous products.

## 4. Conclusions

An effective green approach for the manufacturing of stable anatase CS-TiO_2_ NPs was established using *cannabis sativa* extract as a reducing/stabilizing agent. The XRD peaks of synthesized CS-TiO_2_ are a good match for the anatase phase, which has an average crystallite size of 8 nm. The average particle size distribution of the individual particles was found to be in the range of 12.5 ± 1.5 nm, whereas the average size of the CS-TiO_2_ NP aggregates is 24.5 ± 11.5 nm. The polycrystalline nature of the synthesized green CS-TiO_2_ is confirmed by HR-TEM. In the UV-Vis spectra, CS-TiO_2_ has a plasmon peak around 292 nm with an E_g_ of 4.2 eV. The Ti-O bond stretching at 685 cm^−1^, as well as a variety of functional moieties, were observed using FTIR. Under solar light, nanoparticle catalyzed photolysis of MB dye demonstrated improved degradation efficiency and apparent rate constant when the catalyst dose was increased. Furthermore, the produced CS-TiO_2_ NPs showed outstanding photocatalytic degradation capability against MB dye under sunlight, with a degradation efficiency of 98.2% and an apparent rate constant of 0.0398 min^−1^. As a result, this work adds to the advancement of green photocatalysts.

## Figures and Tables

**Table 1:** Photo-degradation efficiency of CS-TiO_2_ NPs with rate constant.

Catalyst Dose	Degradation Efficiency (%)	Time of Degradation (min)	Apparent Rate Constant (k)	R^2^
5 mg	92.2	100	0.0141 min^−1^	0.991
10 mg	98.2	80	0.0398 min^−1^	0.995

**Table 2:** The photocatalytic performance of CS-TiO_2_ NPs relative to previously reported TiO_2_-based photocatalysts [35,36,37,38,39,40,41,42,43,44,45,46].

Synthesis Method	Catalyst and Dye Concentrations	Radiations	Photocatalytic Degradation	Ref.
Green	10 mg/50 mL (10 mg/L, alizarin red dye)	Sunlight	74%/180 min	[40]
Green	(5 mg/100 mL, corallene red dye)	Sunlight	93%/140 min	[41]
Hydrothermal	10 mg/L (1 mg/L, methylene orange)	Visible	55%/180 min	[42]
Chemical	10 mg/200 mL (methylene blue)	UV	64%/75 min	[43]
Chemical	(methylene blue)	Visible	65%/120 min	[44]
Bio-mediated	100 mg (100 mL, methylene blue)	UV–Visible irradiation	92%/120 min	[28]
Green	10 mg (10 ppm, methylene blue)	UV	96%/120 min	[45]
CS-TiO_2_ NPs	10 mg/L (5 mM, methylene blue)	UV irradiation	98.2%/80 min	This work

## Data Availability

Not applicable.
